# Curcumin promotes ferroptosis in hepatocellular carcinoma via upregulation of ACSL4

**DOI:** 10.1007/s00432-024-05878-0

**Published:** 2024-09-23

**Authors:** Yulang Jiang, Dengcheng Hui, Ziyang Pan, Yongxin Yu, Lu Liu, Xiaofan Yu, Chao Wu, Mingyu Sun

**Affiliations:** 1 Shuguang Hospital Affiliated to Shanghai University of Traditional Chinese Medicine, Shanghai, 201203 China; 2 Shanghai University of Traditional Chinese Medicine, Shanghai, 201203 China; 3 Key Laboratory of Liver and Kidney Diseases, Institute of Liver Diseases, Shuguang Hospital Affiliated to Shanghai University of Traditional Chinese Medicine, No.528 Zhangheng Road, Zhangjiang Hi-Tech Park, Shanghai, 201203 China

**Keywords:** Curcumin_1_, Ferroptosis_2_, Hepatocellular carcinoma _3_, ACSL4_4_, Lipid peroxidation_5_

## Abstract

**Background:**

Ferroptosis, a novel iron-ion-dependent metabolic cell death mode with lipid peroxides as the main driving substrate, plays an irreplaceable role in the development and preventive treatment of hepatocellular carcinoma. Curcumin has potent pharmacological anti-tumor effects.

**Aim of the study:**

We aimed to evaluate the ex vivo and in vivo cancer inhibitory activity of curcumin and its specific mechanism of action.

**Materials and Methods:**

We used the hepatocellular carcinoma cell lines HepG2 and SMMC7721 to assess the direct inhibition of hepatocellular carcinoma proliferation by curcumin in vitro and a tumor xenograft model to evaluate the in vivo cancer inhibitory effect of curcumin.

**Results:**

In this study, we found that ferroptosis’s inhibitors specifically reversed the curcumin-induced cell death pattern in HCC. After curcumin intervention, there was a substantial increase in MDA levels and iron ion levels, and a decrease in intracellular GSH levels. Meanwhile, the expression of GPX4 and SLC7A11 was significantly reduced at the protein levels, while ACSL4 and PTGS2 expression was significantly increased.

**Conclusions:**

This study showed that curcumin significantly decreased the proliferation of HCC cells and significantly increased the sensitivity of ferroptosis. These results suggest that ACSL4 is a viable target for curcumin-induced ferroptosis in treating HCC.

**Supplementary Information:**

The online version contains supplementary material available at 10.1007/s00432-024-05878-0.

## Introduction

Hepatocellular carcinoma (HCC) accounts for more than 80% of all primary liver cancer cases. It is the third leading contributor to cancer-related deaths and the sixth most commonly occurring cancer worldwide. Many HCC patients have unfavorable prognoses, which can be attributed to late diagnosis and unsatisfactory medication efficacy (Siegel et al. 2023).

Ferroptosis is a metabolic mode of cell death distinct from apoptosis, autophagy, and necrosis, characterized by iron ion dependence, lipid peroxide deposition, and an imbalance in the antioxidant system (Dixon et al. 2012). Increased levels of ferrous ions from liable iron pools, peroxidation of the polyunsaturated fatty acid phosphatidylethanolamine, and imbalances in enzyme-dependent and non-enzyme-dependent antioxidant systems induce cellular ferroptosis. Ferroptosis plays an important role in the therapeutic field of HCC (Chen et al. 2021a). Many first-line drugs for the clinical treatment of HCC exhibit strong ferroptosis effects, sorafenib, which shows a strong correlation between drug resistance and ferroptosis sensitivity (Bai et al. 2019). Therefore, the development of hepatocellular carcinoma therapeutic drugs targeting ferroptosis has a broad development prospect (Jiang et al. 2024).

Long-chain acyl coenzyme synthase-4 (ACSL4) is a member of the ACSL family involved in the biosynthesis and catabolism of fatty acids. ACSL4 catalyzes the synthesis of arachidonic acid (AA) into arachidonic acid coenzyme A (AA-CoA) (Feng et al. 2021), AA-CoA generates PUFA-LOOH in the presence of subsequent LPCAT3 enzymes of the ALOX family (Dixon et al. 2015), of which participates in the synthesis of membrane phospholipids, one of the major substrates of ferroptosis membrane damage. Studies have shown that ACSL4 expression is aberrant in clinical cancers, and some of these expression aberrations correlate with poor patient survival. ACSL4 is a very important biological factor in the process of ferroptosis, and ACSL4 can serve both as a biomarker of ferroptosis and as a possible therapeutic target for ferroptosis-related diseases. Therefore, the study of functional changes induced by changes in ACSL4 expression by drug intervention is of great significance for the diagnosis and treatment of HCC.

Many traditional Chinese medicines and their active ingredients have shown better antitumor efficacy (Wu et al. 2022). Compared with traditional Western medicines, Chinese medicines have the advantages of multi-targeting, low toxicity and side effects, and a wide range of safe concentrations. The herbs also contain a variety of active ingredients with regulatory effects on ferroptosis, which has great potential to be developed into an anti-tumor with ferroptosis as a target effect (Wang et al. 2019). *Curcuma longa L.* (turmeric) has the efficacy of activating blood and Qi circulation and relieving pain, which was first recorded in *Tang materia medica*(Tang Dynasty, 659). Curcumin, a polyphenolic compound derived from turmeric, has a variety of pharmacologic effects, including anti-inflammatory, antioxidant, antiproliferative, and antiangiogenic activities (Bai et al. 2022a). Previous studies have shown that curcumin can inhibit the proliferation, invasion, metastasis, and neovascularization of HCC cells (Zhou et al. 2020; Li et al. 2023; Bai et al. 2022b). The mechanism possibly includes inducing apoptosis of tumor cells, increasing the proportion of CD8 + cells to regulate immune function, and inhibiting angiogenesis and lymphoid neogenesis (Guo et al. 2021).

However, the precise mechanism of action and the connection between curcumin and ferroptosis are still unknown. This study aims to investigate how curcumin mediates the occurrence of ferroptosis in HCC.

## Materials and methods

### Cell culture

HepG2 and SMMC7721 Cells were purchased from the Chinese Academy of Sciences Cell Bank (Shanghai, China). The cells were cultured in DMEM (Gibco, #11960-044) supplemented with 10% fetal Bovine serum (Gibco, #10099141) and 1% penicillin and streptomycin (Gibco, #15070-063). All the cells were maintained with 5% CO2 in a 37 °C incubator.

### In vitro cell proliferation assays

The abilities of proliferation of HCC cells in vitro were evaluated by Cell Counting Kit-8 (CCK-8), colony formation, LDH assay, and Calcein/PI assay staining.

For the CCK-8 assay, cells were inoculated into 96-well plates at a density of 3000 cells/well. CCK-8 Reagent (Epizyme Biotechnology Co., Ltd, #CX001) was used to assess cell viability. Briefly, A cell suspension with a cell density of 5000 cells per well was prepared and incubated in a 96 well plate for a specific period. Add 10 μl of CCK8 solution to each well and allow the cck8 reagent to react with viable cells for 4 h. Measure the absorbance of the pores using a microplate reader at a wavelength of 450 nm. The cell viability is calculated by comparing the absorbance values of the treated cells with those of the control cells. Absorbance was measured at 450 nm using an enzyme meter.

For colony formation assay, cells were inoculated in 6-well plates at a density of 100 cells per well and incubated for two weeks in complete medium with different treatment factors. The growing colonies were stained with crystal violet staining reagent (Beyotime Technology Co., Ltd.#C0121). Images were taken with a smartphone and clearly visible colonies (> 20 cells/colony) were counted. The plate clone formation experiment was carried out according to the following steps. A cell suspension with a cell density of 1000 cells per well was cultured in a 6-well plate for 48 h, and corresponding drugs were added for intervention to form colonies. After this, fix and use crystal violet staining solution for cell colony staining. A microscope was used for counting and analyze the form colonies.

Lactate dehydrogenase (LDH)Cytotoxicity Assay Kit (Beyotime Technology Co., Ltd.#C0017), Inoculate the appropriate amount of cells into a 96-well cell culture well plate according to the size and growth rate of the cells, so that the density of cells at the time of the test to be performed does not exceed 80–90% full. Different drugs were added for treatment and appropriate controls were set up. After drug stimulation, the cell culture plate was centrifuged with a multiwell plate centrifuge at 400 g for 5 min. Aspirate the supernatant as much as possible, add 150 μl of LDH release reagent provided in the kit diluted tenfold with PBS (1 volume of LDH release reagent was added to 10 volumes of PBS and mixed, and shake the plate appropriately to mix well and then continue to incubate the plate for 1 h in the cell culture incubator. Subsequently, the cell culture plates were centrifuged in a multiwell plate centrifuge at 400 g for 5 min. 120 μl of the supernatant from each well was taken separately and added to the corresponding well of a new 96-well plate, followed by sample determination and the absorbance of the samples was detected at 490 nm with an enzyme marker.

### Cell migration in vitro

The ability of HCC cells to metastasize in vitro was assessed by the wound scratch test. Cells grown to the filling stage were scratched with a 100 μl pipette tip and incubated in serum-free medium for 12, 24, and 48 h. Images were taken at the beginning and end of the experiment at 100 × magnification.

For Calcein/PI assay (Beyotime Technology Co., Ltd.#C2015), Adherent cells were trypsin-digested and resuspended in culture medium and washed once with PBS; suspended cells were centrifuged at 250–1000 × g for 5 min at room temperature, the supernatant was discarded and washed once with PBS. The recommended cell dosage for each sample was 10^6^ cells. For the precipitate of 106 cells from the previous step, add 1 ml of Calcein AM/PI assay working solution and resuspend to a single-cell suspension. incubate for 30 min at 37 °C, protected from light. note: It is necessary to prepare a buffer-only cell sample to be used as a negative control for the flow cytometry assay, which is suitable to maintain the same buffer as that used to prepare the Calcein AM/PI assay working solution. Two additional tubes of cell samples are also prepared with only one dye (Calcein AM or PI) added to each tube for compensatory adjustment of the flow single stain. After incubation, the cells can be directly detected by flow cytometry, or centrifuged at 250–1000 × g for 5 min at room temperature to precipitate the cells, and after aspirating the liquid, 0.5 ml of buffer was added to each sample to resuspend the cells and then detected by flow cytometry.

### Protein isolation and Western blot analysis

Proteins were extracted using RIPA reagent (Epizyme Biotechnology Co., Ltd, #PC103) and protein concentrations were quantified using a BCA reaction kit (Beyotime Technology Co., Ltd.#P0011). Equal amounts of proteins (25-50 μg) were separated by vertical electrophoresis on 10% SDS-PAGE (Beyotime Technology Co., Ltd.#P0015) and then electro transferred onto a PVDF membrane (Thermo-Fisher Scientific, Pittsburgh, PA, USA). The PVDF membranes were incubated in a protein-free rapid containment solution for 15 min, and then the membranes were incubated with primary antibodies ACSL4 (1: 10,000; ABclonal Biotechnology, China#A20414), PTGS2 (1:1000; ABclonal Biotechnology, China#A3560), GPX4 (1: 1000; ABclonal Biotechnology, China#A11243), SLC7A11 (1: 1000; ABclonal Biotechnology, China#), FTH1 (1: 1000; ABclonal Biotechnology, China#A11243), GSS (1: 500. ABclonal Biotechnology, China#A11557), GAPDH (1: 10,000; ABclonal Biotechnology, China#A19056) at 4° overnight. The next day, HRP-labeled secondary antibody (1: 10,000; ABclonal Biotechnology, China# AS014) was added and developed by chemiluminescence with ECL reagent (Epizyme Biotechnology Co., Ltd, # SQ201). And the expression levels of target proteins were analyzed semi-quantitatively by Image J.

### transient transfection

Cells were seeded at 8000 per well in 6-well plates, and the shACSL4 plasmid was transfected using Poly plus transfection reagent, and the medium was replaced with fresh complete medium after 12 h of transfection for subsequent experiments.

### Reagents

Curcumin(HY-N0005, Purity: 98.16%), Ferrostatin-1(HY-100579), Liproxstatin-1 (HY-12726),Z-VAD-FMK (HY-16658B), Necrostatin-1(HY-15760), 3-Methyladenine(HY-19312), Deferoxamine mesylate (HY-B0988) were purchased from MedChemExpress.

### Malondialdehyde (MDA) and glutathione (GSH) assay

Cells were seeded in cell culture plates at 8000 cells per well, and after 24 h of incubation, cell lysates were collected for detection of intracellular MDA (Beyotime Technology Co., Ltd.#S0131) and GSH (Beyotime Technology Co., Ltd.#S0052) content according to the kit instructions. MDA was detected at 535 nm and GSH at 412 nm with an enzyme marker.

### *Iron* assay

For cells, cellular ferrous ion levels were detected with the ferroOrange probe (Dojindo#F374). Cells were seeded at 8000 per well in a six-well plate, cell crawls were made, and after 24 h, the probe was stained for 30 min and washed three times with PBS. Subsequently, the cellular ferrous ion level was observed by laser confocal microscopy. For tissues, 100 mg of tumor tissue was lysed and the supernatant was taken after centrifugation at 12,000 g. Reaction reagents were added sequentially according to the instructions. Add all Standard tubes, sample tubes, and sample blank tubes to a 96-well plate and incubate for 60 min, then measure the absorbance at 593 nm. The total and ferrous iron content of the tissue was also calculated according to the standard curve.

#### Hydrogen peroxide and superoxide detection assay

Intracellular hydrogen peroxide and superoxide levels were used to evaluate the level of intracellular oxidation.

For the hydrogen peroxide assay, cells were seeded in 96-well plates and after 24 h of drug incubation, supernatants were taken and assayed according to the assay kit instructions (Nanjing Jiancheng Bioengineering Institute#A064-1-1).

For the superoxide assay (Beyotime Technology Co., Ltd.#S0060), cells were seeded in 96-well plates and drug interventions were added for 24 h, then the superoxide kit instructions were followed, and the absorbance of the samples was read at 450 nm.

#### Mitochondrial membrane potential assay

Cells were seeded at 5000 per well in 12-well plates, and after different groups were given different drugs for 24 h of stimulation, intracellular mitochondria were localized and stained using Mito-Tracker Red CMXRos (Beyotime Technology Co., Ltd.#S0131), and the mitochondrial membrane potential was observed under an inverted fluorescence microscope, and the average fluorescence intensity was calculated using Image J. The cells were then stained with Mito-Tracker Red CMXRos, and the mitochondrial membrane potential was measured using Mito-Tracker Red CMXRos.

#### Immunohistochemistry

The immunohistochemistry (IHC) procedure involves several steps. First, the tumor tissues were fixed and embedded. Then, it is cut into thin sections measuring 4 μm. Antigen repair was performed in sodium citrate buffer at 95–100 °C for 40 min. To minimize non-specific binding, 5% BSA was used for blocking. The primary antibody was incubated at 4 °C overnight to facilitate specific binding of the target antigen. Subsequently, the secondary antibody, conjugated with a detection molecule, was incubated to bind to the primary antibody. The visualization of target proteins was achieved through the utilization of DAB colorimetric solution. Subsequently, a quantitative analysis of each sample was conducted from three distinct perspectives.

#### Lipid peroxidation assay

Cells were seeded in six-well plates at 8000 per well, and after 24 h, different groups of drugs were given to stimulate for 24 h, liperfluo probe (Dojindo#L248) was incubated for 30 min, PBS washed three times, and then cells were digested by 0.25% EDTA trypsin in flow tubes and detected by flow cytometer on the machine.

#### Animal model

To establish a mouse model of hepatocellular carcinoma xenografts, HepG2 cells were injected subcutaneously into the right abdomen of 5- to 6-week-old nude mice. Approximately 10^7^ cells were injected into each. The maximum width (X) and length (Y) of the tumors were measured with calipers every three days, and the volume (V) was calculated using the following equation. The volume (V) was calculated using the formula: V = (X^2^Y)/2. The mice were randomly divided into two groups: (1) a drug group, in which the mice were injected intraperitoneally with curcumin (20 mg/kg) every day and fed a normal diet; and (2) a control group, in which the mice were injected intraperitoneally with an equal amount of saline and fed a normal diet every day. mice were executed for material collection after 30 days.

All animal experimental protocols were approved by the Animal Center and Use Committee of Shanghai University of Traditional Chinese Medicine.

#### Statistical analysis

All data was shown as mean and Standard Deviation (SD). The Kolmogorov–Smirnov or Shapiro–Wilk test was used to determine if the data were regularly distributed. Comparisons of the significance between two variables assuming they were regularly distributed aired or unpaired two-tailed T tests were used to analyze the groups, and Comparisons of three or more groups' importance were made either a two- or one-way ANOVA. If the distribution of the data were skewed, utilizing comparisons of the importance between two groups, comparisons of significance using the nonparametric Mann–Whitney U test utilizing the Kruskal–Wallis test across three or more groups.

## Results

### Curcumin dose-dependently inhibits HCC cells proliferation and metastasis

**Fig. 1:**
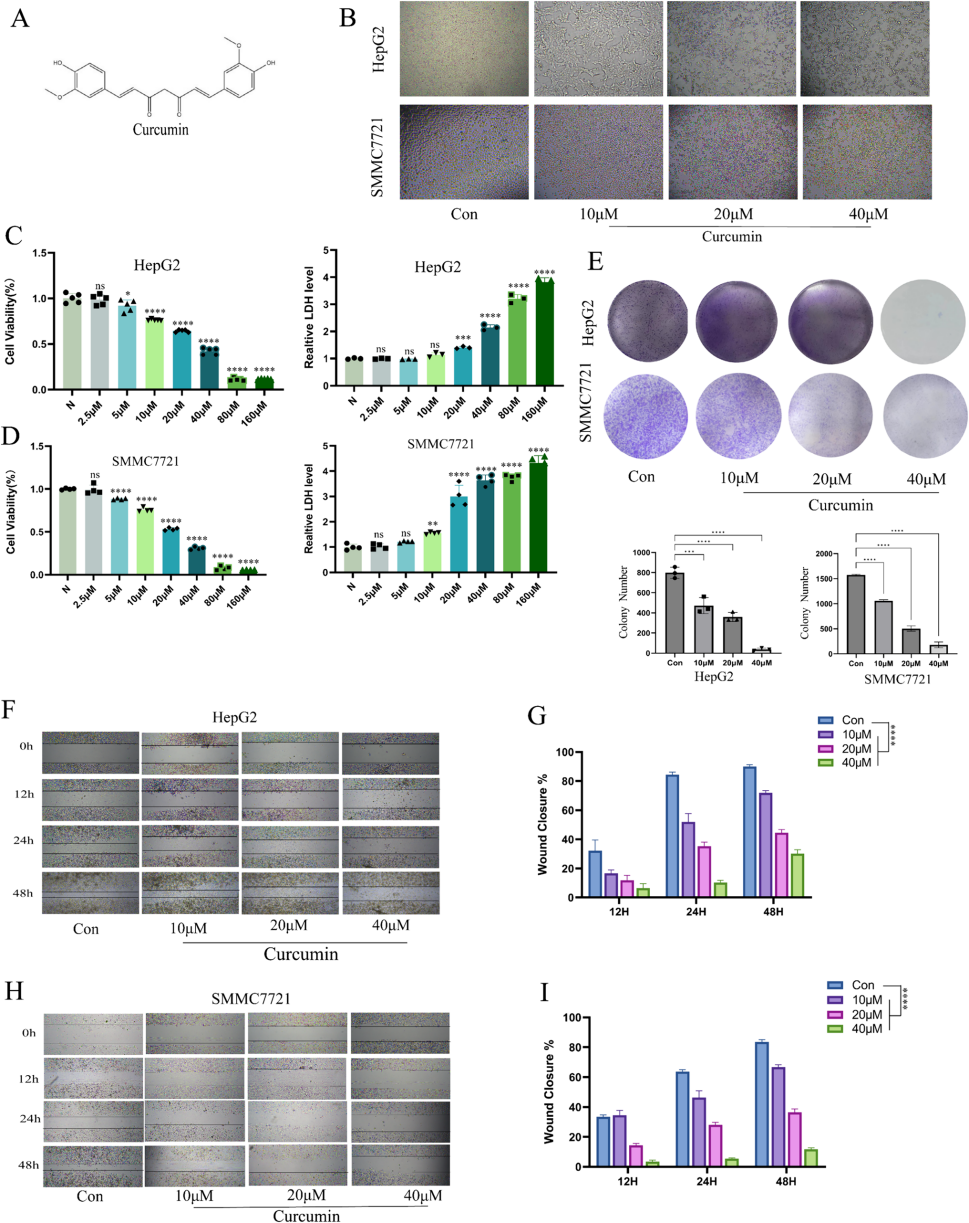
Curcumin inhibits hepatocellular carcinoma cell proliferation and migration in vitro. **A** The Structural formula of curcumin. **B** Morphology of HepG2 cells and SMMC7721 cells in the presence of different concentrations of curcumin. **C**, **D** Curcumin exhibited a dose-dependent cytotoxic effect on HepG2/SMMC7721 and could increase its LDH release in a concentration gradient. **E** Clone formation experiments showed the effect of curcumin on the long-term proliferative capacity of HCC cells. **F**, **I** Representative images and quantitative analysis of the Wound healing assay for treated by Curcumin HepG2/SMMC7721 cells. *P < 0.05, **P < 0.01, ***P < 0.001, ****P < 0.0001

Figure 1A illustrates the molecular formula of curcumin. We first picked HCC cell lines HepG2 and SMMC7721 to evaluate the in vitro antitumor activity of curcumin. After acting on HCC cells with different concentrations of curcumin for 24 h, we observed under the microscope that as the concentration of curcumin action increased, the cell morphology changed, the cells became elongated, the nuclei rounded, the cytoplasmic contents increased and eventually the cell floats away and dies (Fig. 1B). Further to determine the effective drug concentration of curcumin to inhibit HCC cells in vitro, we evaluated cell viability and cytotoxicity by CCK8 assay and LDH release assay after 2.5 μM, 5 μM, 10 μM, 20 μM, 40 μM, 80 μM, and 160 μM of curcumin acted on HepG2 and SMMC7721 24 h. We found that the cell viability of HCC cells was significantly reduced when the concentration of curcumin was 5 μM, and when the concentration of curcumin was increased to 10 μM, the amount of lactate dehydrogenase in the supernatant of the cells was significantly increased (Fig. 1C, D), this means that curcumin produces cytotoxicity. We also found that curcumin had an inhibitory effect on the clone formation of HCC cells in a dose-dependent manner (Fig. 1E). Further, we found that curcumin significantly inhibited the metastatic ability of hepatocellular carcinoma cells HepG2 and SMMC7721 by wound healing experiments when compared with the blank control group (Fig. 1F, I)., but we did not evaluate the toxic effects of curcumin on normal hepatocytes. Although our data suggest that curcumin can dose-dependently inhibit hepatocellular carcinoma cell proliferation, we selected human normal hepatocytes L02 and mouse normal hepatocytes AML12 for cell viability assay using CCK8 assay. The results showed that curcumin had no significant effect on the cell viability of L02 cells at a concentration of 40 μM and below (P > 0.05) and showed inhibition of the cell viability of L02 cells at a curcumin concentration of 80 μM (P < 0.05) (Fig. S1A). Similar results were shown in AML12 cells (Fig. S1B), while we chose curcumin concentrations of 10 μM, 20 μM and 40 μM in the subsequent experiments, so it did not cause significant damage to normal hepatocytes. Further, we examined the changes in liver function in the serum of nude mice under curcumin intervention, and the results showed that curcumin did not impair liver function in nude mice. We also examined the changes of ACSL4 protein and mRNA levels in L02 cells under curcumin intervention, and the results showed that different concentrations of curcumin had no significant effect on the levels of ACSL4 in L02 cells (P > 0.05) (Fig. S1D–F). This seems to suggest an interesting phenomenon that curcumin can specifically target ferroptosis in hepatocellular carcinoma cells, whereas normal hepatocytes seem to have an ferroptosis resistance mechanism to curcumin. This suggests that if curcumin combined with ferroptosis inducers might have a significant synergistic effect, and we might follow up with further studies around this topic. Secondly, ferroptosis as an ancient and conserved metabolic death mode has natural ferroptosis resistance genes in cells and tissues, and finding the regulators or checkpoints of ferroptosis sensitivity among different cells or tissues will be the focus within the field of ferroptosis research in the future.

### Curcumin induces ferroptosis in HCC cells by increasing intracellular MDA levels

**Fig. 2:**
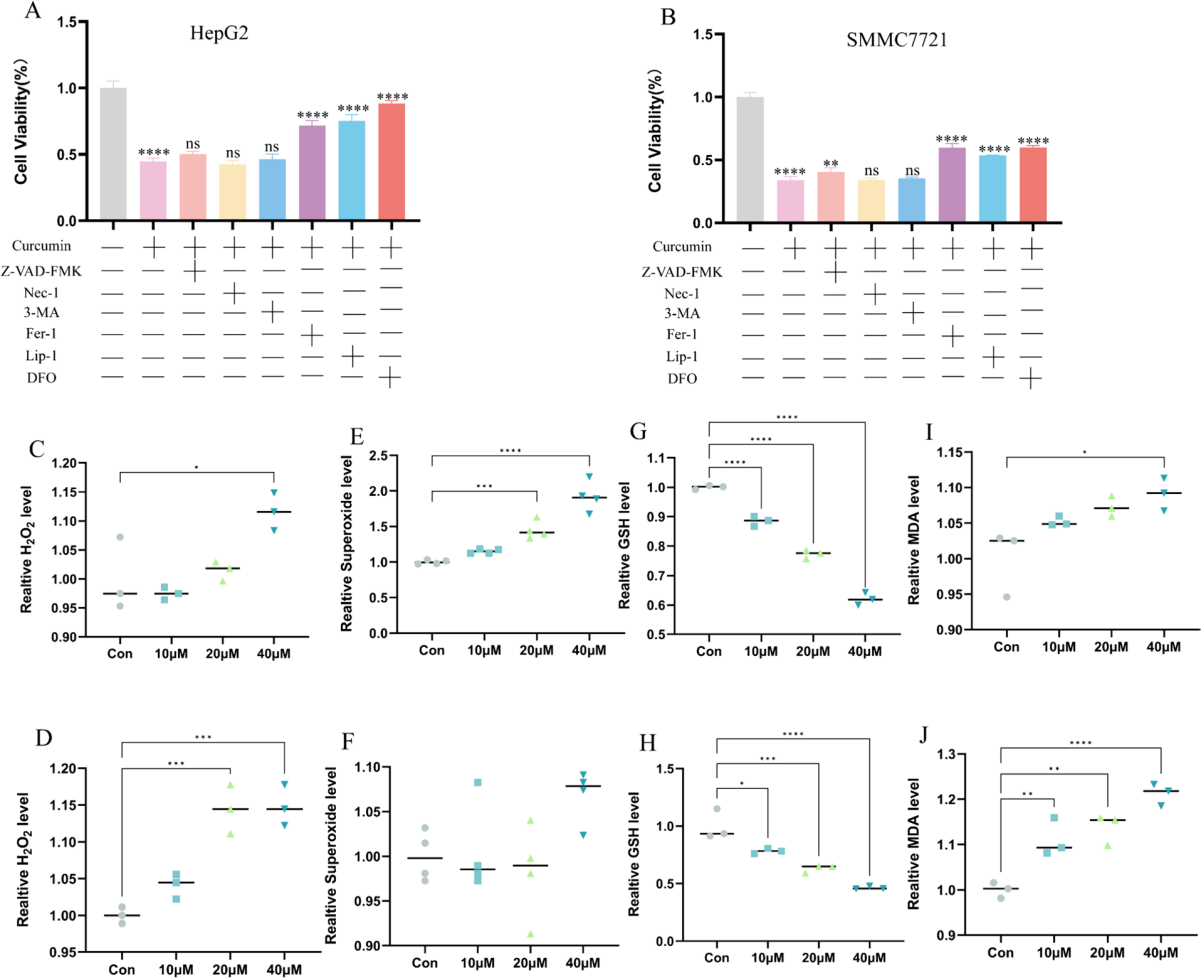
Curcumin causes ferroptosis by increasing MDA level and decreasing GSH level in HCC cells. **A**, **B** The cell death mode caused by curcumin could be specifically rescued by the ferroptosis inhibitors Fer-1, Lip-1 and DFO, but not by the apoptosis inhibitor Z-VAD-FMK, the necrosis inhibitor Nec-1, and the autophagy inhibitor 3-MA. **C**, **D** Hydrogen peroxide levels in HepG2 and SMMC7721 cells after curcumin treatment. **E**, **F** Superoxide levels in HepG2 and SMMC7721 cells after curcumin treatment. **G**, **H** GSH levels in HepG2 and SMMC7721 cells after curcumin treatment **I**, **J** MDA levels in HepG2 and SMMC7721 cells after curcumin treatment. *P < 0.05, **P < 0.01, ***P < 0.001, ****P < 0.0001

In order to explore the mode of curcumin-induced cell death, we used different inhibitors of programmed cell death, and we were pleasantly surprised to find that HCC cell viability was partially rescued only after the specific use of the ferroptosis inhibitors Fer-1, Lip-1, and DFO, whereas inhibitors of apoptosis, necrosis and autophagy were not able to rescue the HCC cells from death (Fig. 2A, B). This suggests that curcumin-mediated cell death in HCC may be dominated by ferroptosis. Increased intracellular lipid peroxidation and diminished antioxidant capacity are important hallmark events in the development of ferroptosis (Stockwell 2022). Therefore, we examined the intracellular levels of hydrogen peroxide, superoxide, GSH, and MDA in HepG2 and SMMC7721 separately. We found that the oxidative and antioxidant balance of hepatocellular carcinoma cells was disrupted after curcumin intervention. The levels of H2O2 and superoxide did not significantly change(Fig. 2C–F). In contrast, GSH levels were significantly decreased (Fig. 2G, H) and MDA (Fig. 2I, J) content was significantly increased in cell lysates. Similar results were obtained in both HepG2 and SMMC7721 hepatocellular carcinoma cell lines. These results indicate that curcumin can induce ferroptosis in hepatocellular carcinoma cells by modulating intracellular GSH and MDA levels.

### Curcumin upregulates ACSL4 in HCC cells to induce ferroptosis

**Fig. 3:**
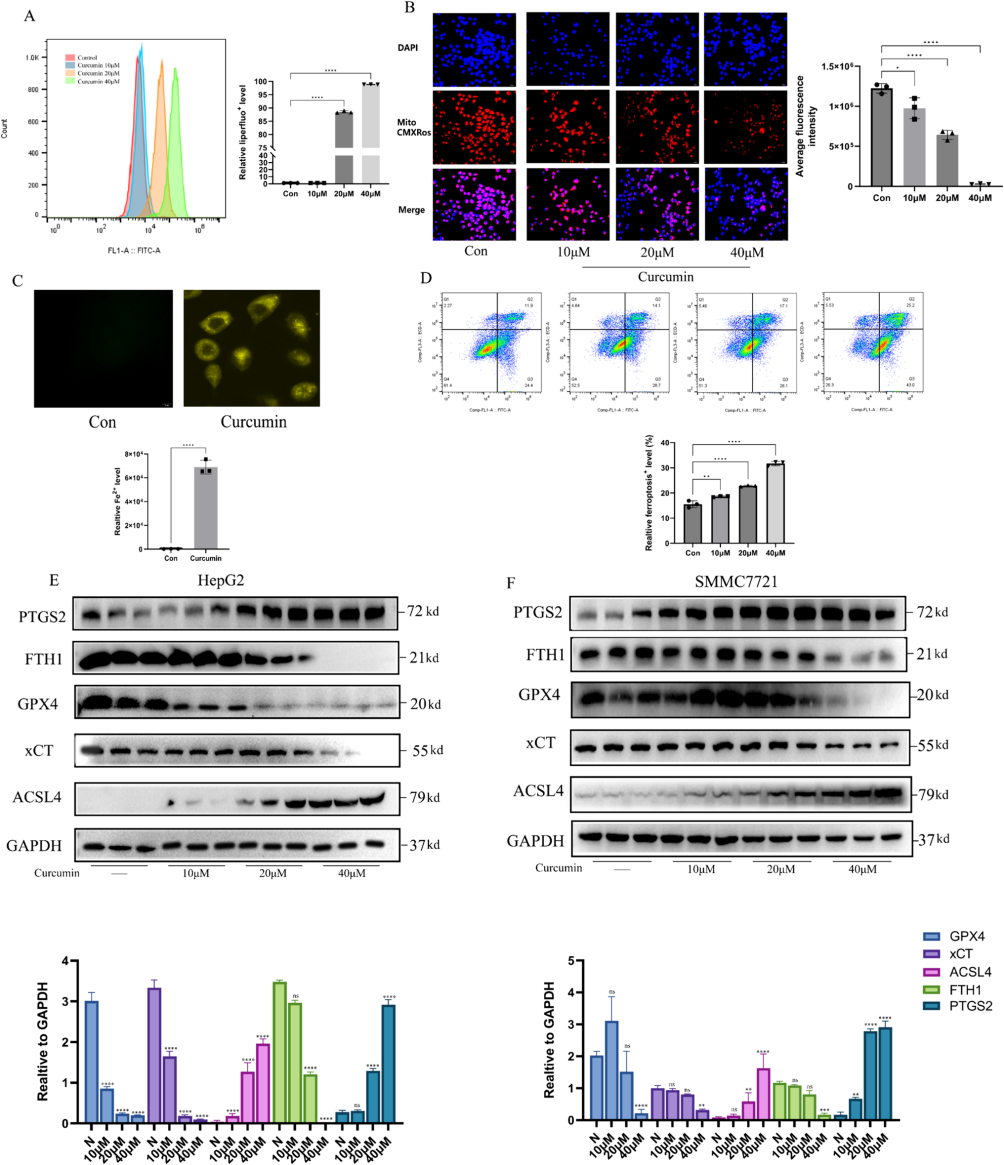
Curcumin induces ferroptosis in HCC cells. **A** Curcumin can dose-dependently increase the level of lipid peroxides in HCC cells. **B** Curcumin can dose-dependently reduces mitochondrial membrane potential in HCC cells. **C** Curcumin increases ferrous ion levels in HCC cells. **D** Curcumin can dose-dependently increase the proportion of ferroptosis in HCC cells. (E–F) Curcumin-stimulated expression levels of ferroptosis-related proteins were detected by WB in HCC cells. *P < 0.05, **P < 0.01, ***P < 0.001, ****P < 0.0001

To further evaluate the intracellular metabolic status of hepatocellular carcinoma cells under curcumin stimulation, we detected the intracellular lipid peroxides levels using a liperfluo probe, and the intracellular lipid peroxides levels were significantly increased when HepG2 cells were stimulated with 20 μM curcumin for 24 h (Fig. 3A) (*P* < 0.0001). Moreover, curcumin could reduce the mitochondrial membrane potential level of HepG2 cells in a concentration gradient (Fig. 3B). Iron ion metabolism in tumor cells is closely linked to the level of ferroptosis, especially the content of divalent ferrous ions in liable iron pools. High ferrous ion levels significantly increase tumor cell ferroptosissensitivity. Significantly increased levels of ferrous ions were detected in HepG2 cells when exposed to curcumin, as shown by the ferroOrange probe (Fig. 3C). Calcein-AM (calcein acetoxymethyl ester) is a non-fluorescent, cell-penetrating dye that emits intense green fluorescence after hydrolysis catalyzed by the enzyme oleoresterase and conversion to calcein in cells. Because it only stains living cells and has very low cytotoxicity, the fluorescence intensity is proportional to the viability of the cells to a certain extent, so it is commonly used in cell viability assays to distinguish between living and dead cells. We found that the proportion of hepatocellular carcinoma cells undergoing ferroptosis showed a gradual increase as the curcumin-stimulated concentration increased (Fig. 3D). In order to deeply investigate which process of ferroptosis curcumin is involved in, we examined the classical regulators of ferroptosis process by Western Blot, and found that curcumin can be widely involved in various processes of ferroptosis, which includes the decrease of ferritin heavy chain 1 (FTH1) level, This may be the reason for the increase of intracellular ferrous ions after curcumin action (Kong et al. 2021). At the same time, the activity of the SLC7A11/GPX4 axis, an anti-ferroptosis system in hepatocellular carcinoma cells, was also significantly inhibited by curcumin, which further exacerbated the degree of intracellular ferroptosis in hepatocellular carcinoma cells. We also found increased levels of PTGS2, a biomarker of ferroptosis. In view of a previous article reporting a regulatory effect of curcumin on ACSL4 protein, we also examined ACSL4 levels under curcumin intervention in HCC cells, and found that the protein level of ACSL4 was significantly increased (Fig. 3F, G). This could potentially cause an increase in lipid peroxidation, which is a precursor to ferroptosis in HCC. Due to the significant regulatory effect of curcumin on ACSL4 protein levels in HCC cells, we have proposed the hypothesis that curcumin may promote ferroptosis in HCC and thus inhibit HCC proliferation by up-regulating ACSL4 levels.

### Knockdown of ACSL4 rescued ferroptosis caused by curcumin

**Fig. 4:**
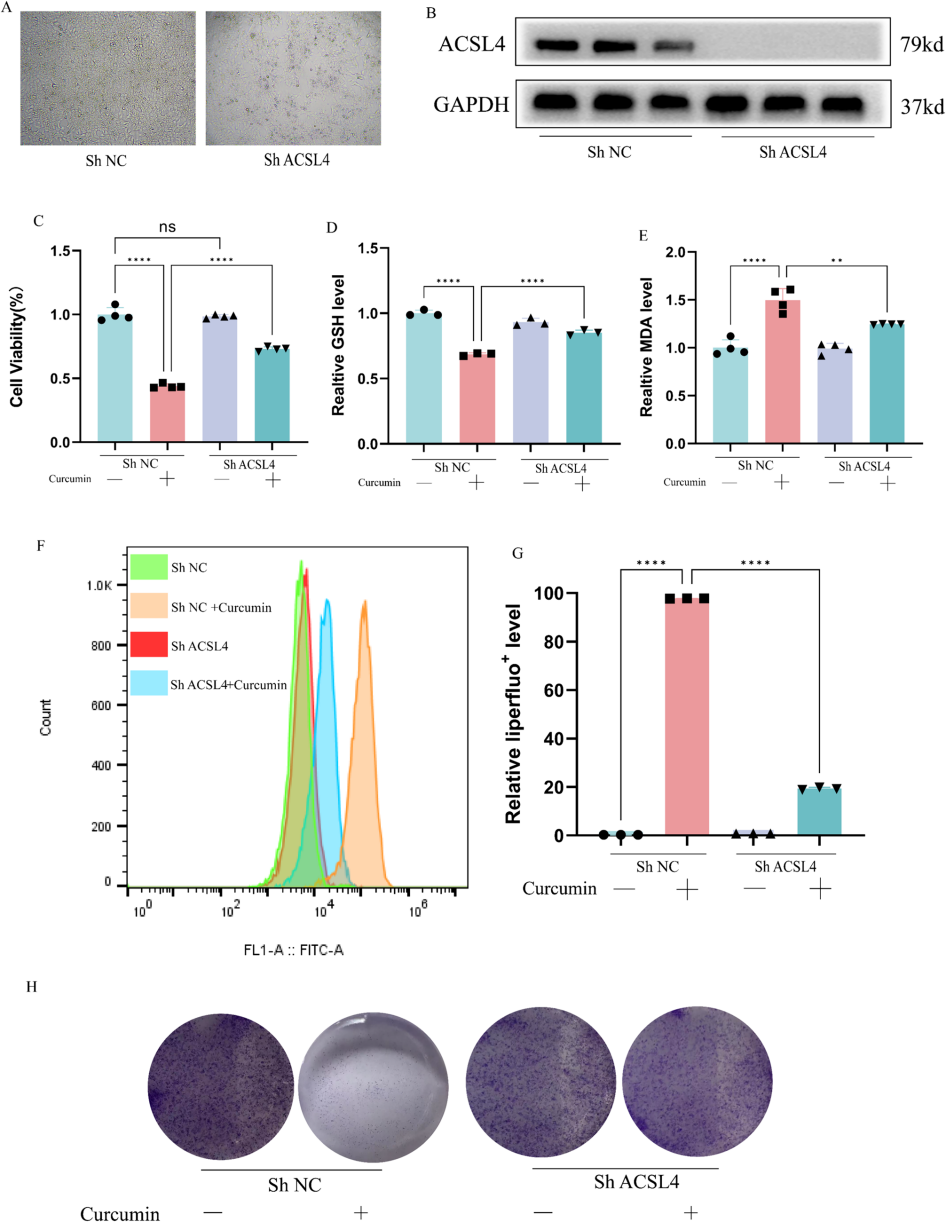
Knockdown of ACSL4 inhibits curcumin-induced ferroptosis in HCC cells. **A** Morphological changes in HepG2 cells after knockdown of ACSL4. **B** Protein expression levels of ACSL4 after knockdown of ACSL4. **C**–**E** Relative levels of HepG2 cell viability, GSH, MDA under curcumin intervention after knockdown of ACSL4. **F**, **G** Changes in the levels of lipid peroxides in HepG2 cells under curcumin intervention after knockdown of ACSL4 and its semi-quantitative analysis. **H**–**J** Clone formation experiment under curcumin intervention after knockdown of ACSL4. *P < 0.05, **P < 0.01, ***P < 0.001, ****P < 0.0001

To verify that ACSL4 is a core target of curcumin-mediated ferroptosis, we constructed ACSL4-knockdown HepG2 cells, and there was no significant change in cell status and viability after transfection with the sh ACSL4 plasmid (Fig. 4A), and the level of ACSL4 protein was markedly reduced compared with the Vector group (Fig. 4B). After stimulation of HepG2 cells with 40 μM curcumin for 24 h, We examined the viability of HCC cells and found that the viability of hepatocellular carcinoma cells was not significantly reduced after knockdown of ACSL4, but the inhibitory activity of curcumin against HepG2 was significantly attenuated after knockdown of ACSL4 (Fig. 4C). Further we also examined the level of intracellular oxidative stress under the effect of curcumin after knockdown of ACSL4. Curcumin-induced knockdown of ACSL4 resulted in a bright increase in GSH levels (Fig. 4D) and a significant decrease in MDA levels (Fig. 4E), while the liperfluo probe showed the same trend (Fig. 4F, G). These results suggest that the knockdown of ACSL4 can reply to curcumin-induced hypersensitivity to ferroptosis in hepatocellular carcinoma cells. Further we examined the protein expression levels of SLC7A11 and GPX4, key genes for ferroptosis. The clone formation assay also reflected the attenuation of curcumin's ability to inhibit long-term proliferation of hepatocellular carcinoma cells after knockdown of ACSL4 (Fig. 4H).This suggests that the level of defense against ferroptosis in hepatocellular carcinoma cells was significantly increased after silencing ACSL4 and mainly involved the cystine/glutathione/glutathione peroxidase 4 signaling pathway axis. This suggests that ACSL4 may be the core target of curcumin-mediated ferroptosis in hepatocellular carcinoma cells.

### Curcumin inhibits tumor growth of cell xenografts in nude mice

**Fig. 5:**
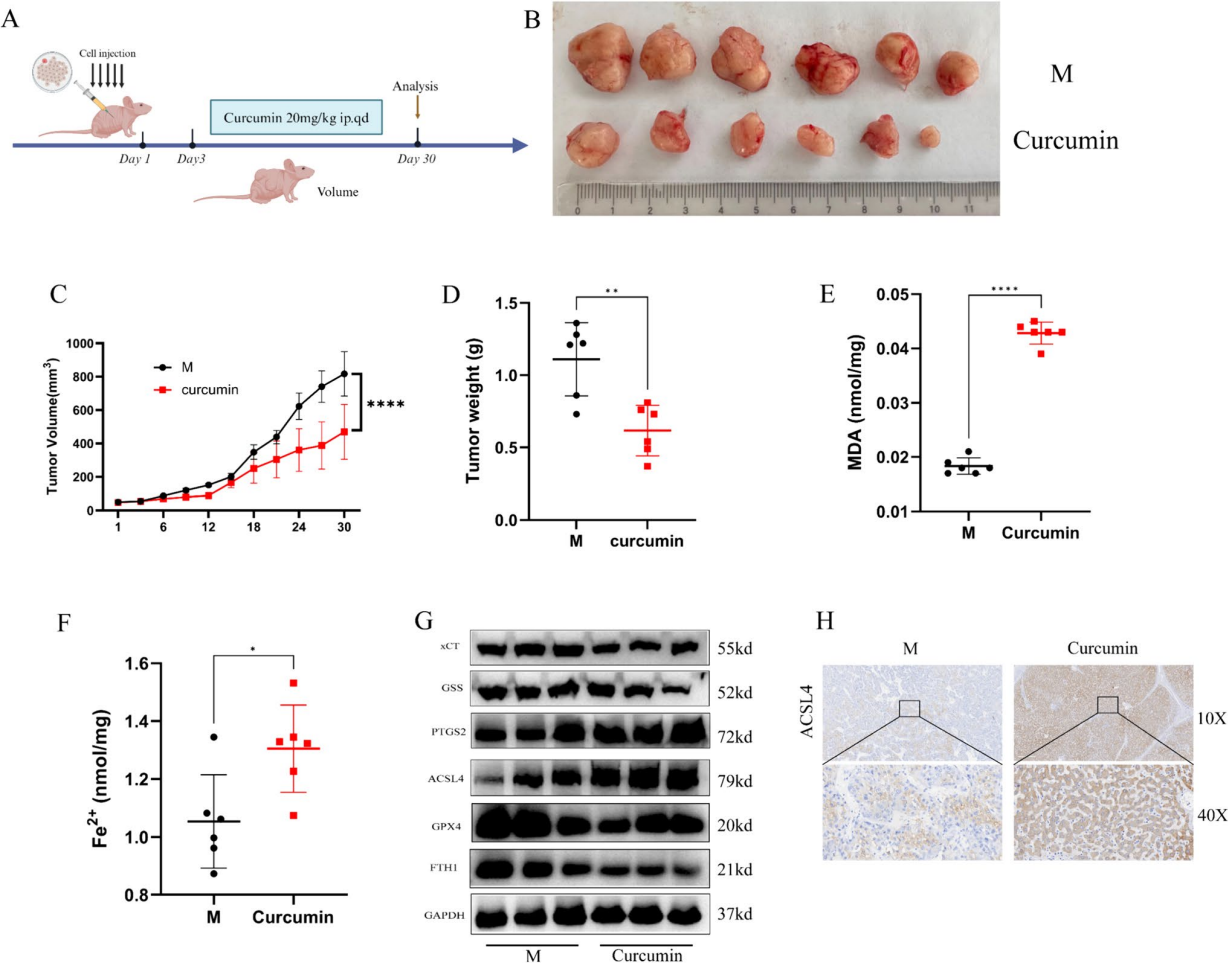
Curcumin can induce ferroptosis in HCC cells in vivo. **A** Schematic representation of the experimental design for mice. **B** Gross appearances of liver tumors. **C**, **D** Tumor weight (**C**) and tumor growth curves (**D**) in the nude mouse xenograft model. **E**, **F** MDA and Fe^2+^ content production in tumor tissues. **G** Expression levels of ferroptosis-related proteins in HCC tissues by WB. **H** Expression of ACSL4 by immunohistochemistry in paraffin sections of HCC tissues. *P < 0.05, **P < 0.01, ***P < 0.001, ****P < 0.0001

**Fig. 6:**
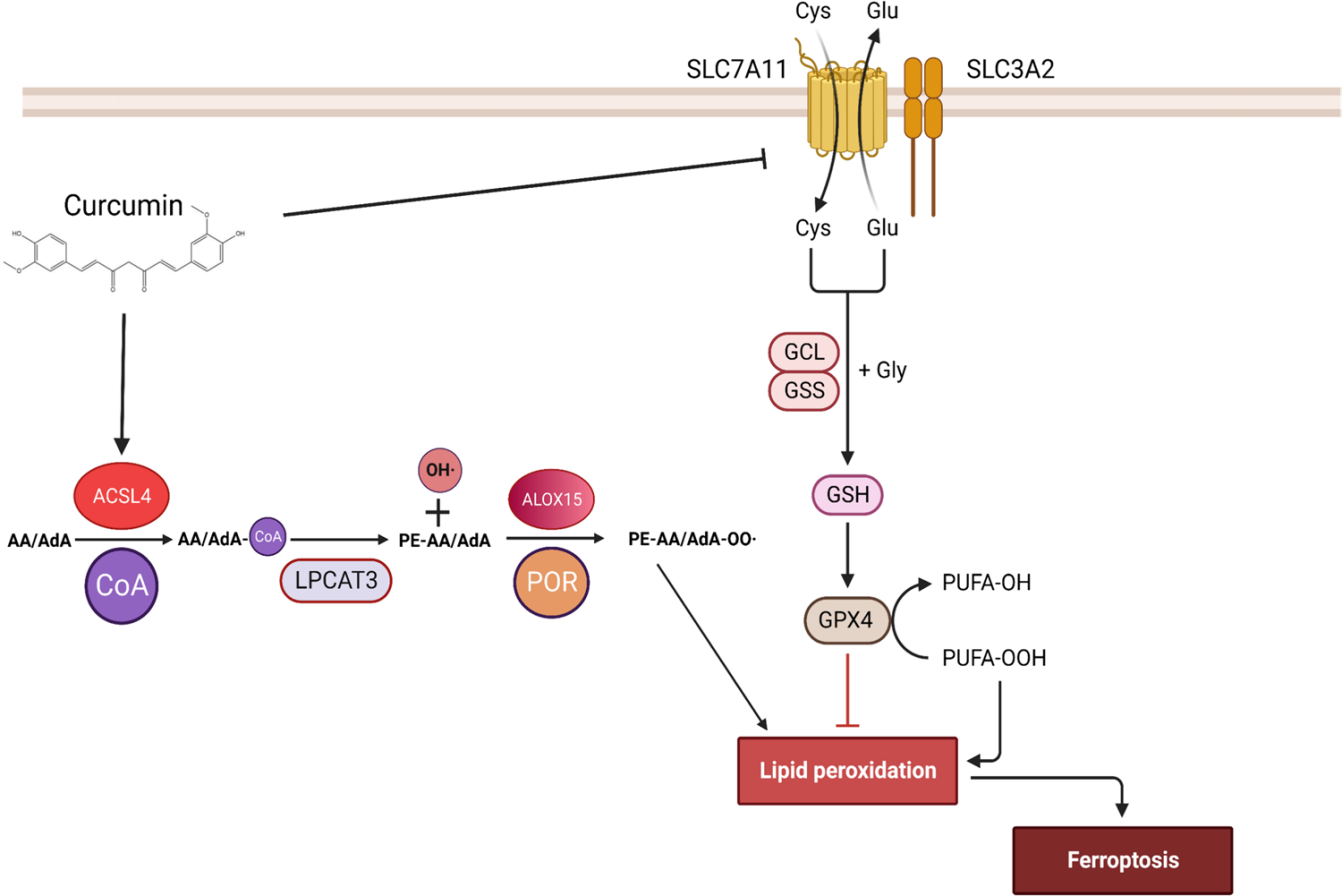
Schematic diagram of the mechanism of curcumin-induced ferroptosis in hepatocellular carcinoma. Curcumin can up-regulate the expression of ACSL4 in hepatocellular carcinoma cells and promote the production of PUFA-PE. On the other hand, curcumin can inhibit the expression of SLC7A11, which reduces the production of GSH in hepatocellular carcinoma cells, making the cellular antioxidant capacity weakened, and ultimately resulting in the accumulation of lipid peroxides in hepatocellular carcinoma cells, which leads to the occurrence of ferroptosis

To investigate whether curcumin has a clear ferroptosis effect in hepatocellular carcinoma in vivo, we used a model of subcutaneous tumor formation in nude mice. After acclimatization feeding of nude mice, HepG2 cells were injected into the right axilla of nude mice at a density of 8 million each. Three days after subcutaneous injection of HepG2, curcumin was administered to mice by gavage at a dose of 20 mg/kg every day, and the body weights of nude mice and the volume of tumors were recorded every three days, Samples were taken and analyzed after 30 days (Fig. 5A). The results of in vivo experiments showed that curcumin significantly reduced tumor volume and tumor weight (Fig. 5B, D). Curcumin inhibited the proliferation rate of hepatocellular carcinoma as seen in the time-volume curves of tumors (Fig. 5C). At the same time, curcumin also increased MDA and Fe^2+^ content in liver cancer tissues (Fig. 5E, F). The results of Western Blot showed that curcumin decreased the expression of ferroptosis-related proteins SLC7A11, GSS, GPX4, and FTH1, as well as increased the expression levels of PTGS2 and ACSL4 in hepatocellular carcinoma tissues (Fig. 5G). Immunohistochemistry also showed increased levels of ACSL4 (Fig. 5H). In conclusion, all of the above results suggest that curcumin can inhibit hepatocellular carcinoma proliferation and growth by up-regulating the level of ferroptosis in hepatocellular carcinoma tissues (Fig. 6).

## Discussion

HCC is still a problem for the world's health, and its prevalence is rising globally. It is predicted that by 2025, more than 1 million people would be impacted by liver cancer every year. With over 90% of cases, HCC is the most prevalent kind of liver cancer (Huang et al. 2020). Current liver cancer treatment mainly includes surgery, chemotherapy, targeted therapy, immunotherapy, etc. (Liu et al. 2022). Although the progression-free survival of patients has been prolonged with the prescription of new generation of targeted drugs, the help to patients is still very limited (Huo et al. 2021). With the discovery of more and more anti-cancer Chinese medicines and their active ingredients (Li et al. 2021), the quality of life of liver cancer patients has been greatly improved as well as the dignity of advanced patients (Gao et al. 2021). Curcumin is an anti-tumor active ingredient extracted from the traditional Chinese medicine turmeric, curcumin has inhibitory effect on the proliferation of many kinds of tumors, and studies have shown that curcumin can be used in the treatment of breast cancer through the ROS-YAP-JNK pathway (Guo et al. 2021). Curcumin can be used to inhibit the activation of JAK/STAT3 signaling pathway in GC (Ham et al. 2022), thus overcoming the resistance to chemotherapy in gastric cancer. Curcumin can activate ROS/KEAP1/NRF2/miR-34a/b/c cascade to inhibit colorectal cancer metastasis (Liu et al. 2023). Curcumin enhances the therapeutic effect on NSCLC by activating the autophagy-induced ferroptosis response in NSCLC (Chen et al. 2019).

In the treatment of hepatocellular carcinoma, curcumin reduced the level of alpha-fetoprotein in liver tissue, increased the number of immune cells (e.g., NK cells), and inhibited EMT by regulating IL-6/JAK/STAT3 and IL-1β/NF-κB pathways, thereby protecting against the progression of hepatocellular carcinoma (Man et al. 2020). Curcumin down-regulated BCLAF1 expression, inhibited activation of the PI3K/AKT/GSK-3β pathway, and triggered mitochondrial apoptosis in HCC (Bai et al. 2022a). Curcumin inhibited the proliferation, migration and invasion of HCC cells by regulating miR-21-5p and SOX6, indicating that curcumin has a favorable pharmacological effect on HCC (Zhou et al. 2020).

ACSL4 plays an important role in ferroptosis-related lipid metabolism, especially its mediated activation of arachidonic acid and adrenoic acid, which are the key substrates and rate-limiting steps of ferroptosis.ACSL4 can be used both as a biomarker of ferroptosis sensitivity and a possible therapeutic target for the treatment of iron-death-related diseases (Lee et al. 2021). Meanwhile, ACSL4 appears to be abnormally expressed in a variety of clinical cancers, and ACSL4 overexpression in hepatocellular carcinoma is often predictive of poor prognosis related to HCC patients (Feng et al. 2021). But surprisingly, the high expression of ACSL4 in HCC implies that hepatocellular carcinoma is highly sensitive to ferroptosis, and thus inducing hepatocellular carcinoma cells to target ferroptosis is a new direction for precision tumor therapy (Grube et al. 2022).

Based on the high expression of ACSL4 in hepatocellular carcinoma, this implies that ACSL4 may be a key gene for ferroptosis resistance in hepatocellular carcinoma. We found in the TCGA database that ACSL4 was significantly higher in HCC tissues than in normal tissues (Dai et al. 2022). Also in vivo and in vitro experiments verified that curcumin could induce ferroptosis. It has been shown that ACSL4 plays an important role in ferroptosis and ACSL4 is a key enzyme in lipid peroxidation generation. Our study also found that curcumin significantly up-regulated ACSL4.In normal cells, the Xc- system acts as a cystine/glutamate transporter that transports cysteine molecules into the cell in exchange for glutamate molecules in the cell.SLC7A11 is an important component of the Xc- system (Chen et al. 2021b).Cystine absorbed by SLC7A11 into the cell is converted by the reduction reaction of depleting NADPH to SLC7A11 is an important component of the Xc- system (Koppula et al. 2021). Our study found that curcumin acts on SLC7A11 to reduce its expression, which leads to a decrease in the amount of cystine transported into the cell, resulting in a decrease in intracellular GSH levels, which in turn effectively inhibits the iron apoptotic function of GPX4, ultimately leading to ferroptosis of tumor cells.

However, the specific molecular mechanisms remain to be further investigated. In addition, small molecule compounds targeting ACSL4 have been used in the treatment of tumors. In summary, our study found that curcumin can increase the expression of ACSL4, leading to an increase in MDA production, and curcumin can also reduce the level of SLCA711, leading to a decrease in GSH production, which further affects the activity of GPX4 and ultimately leads to the occurrence of ferroptosis response. We discovered a new molecular mechanism of curcumin, an active ingredient of traditional Chinese medicine, to inhibit the proliferation of hepatocellular carcinoma, which provides a more in-depth and scientific explanation for the treatment of tumors by traditional Chinese medicine.

A number of studies on curcumin anticancer have been accumulated, and it is currently believed that curcumin can effectively induce apoptosis in hepatocellular carcinoma cells, but whether curcumin is involved in the regulation of other programmed cell death modalities remains unknown, and the crosstalk between different cell death modalities has not been elucidated. Our study preliminarily explored the potential of curcumin as a novel ferroptosis inducer and its mechanism of action. Meanwhile, the poor oral utilization and bioactivity of curcumin are responsible for its low blood concentration and short duration of action. The current research on nano drug delivery systems seems to be promising to provide oral utilization and bioactivity of curcumin, and in the future, multidisciplinary cross-fertilization and cooperation between medical, technological and industrial disciplines need to be actively sought to solve the current dilemma of clinical application of curcumin.

## Conclusions

The discovery of in vivo active ferroptosis-inducing herbal monomers is extremely important for the complementary treatment of clinical tumors. In conclusion, our study reveals a new mechanism of curcumin against hepatocellular carcinoma and identifies the core target of curcumin-mediated ferroptosis, ACSL4, which provides a broader scientific explanation for the clinical application of curcumin.

## Supplementary Information

Below is the link to the electronic supplementary material.

Supplementary file1 (DOCX 950 KB)

## Data Availability

The datasets used and/or analyzed during the current study are available from the corresponding author on reasonable request.
