# Supplementary material for: Curcumin promotes ferroptosis in hepatocellular carcinoma via upregulation of ACSL4
Source: J Cancer Res Clin Oncol. 2024 Sep 23;150(9):429. doi: 10.1007/s00432-024-05878-0 (PMC11420324; doi:10.1007/s00432-024-05878-0)
Supplement: Supplementary file 1 — Supplementary file1 (DOCX 950 KB) [file 432_2024_5878_MOESM1_ESM.docx]

**
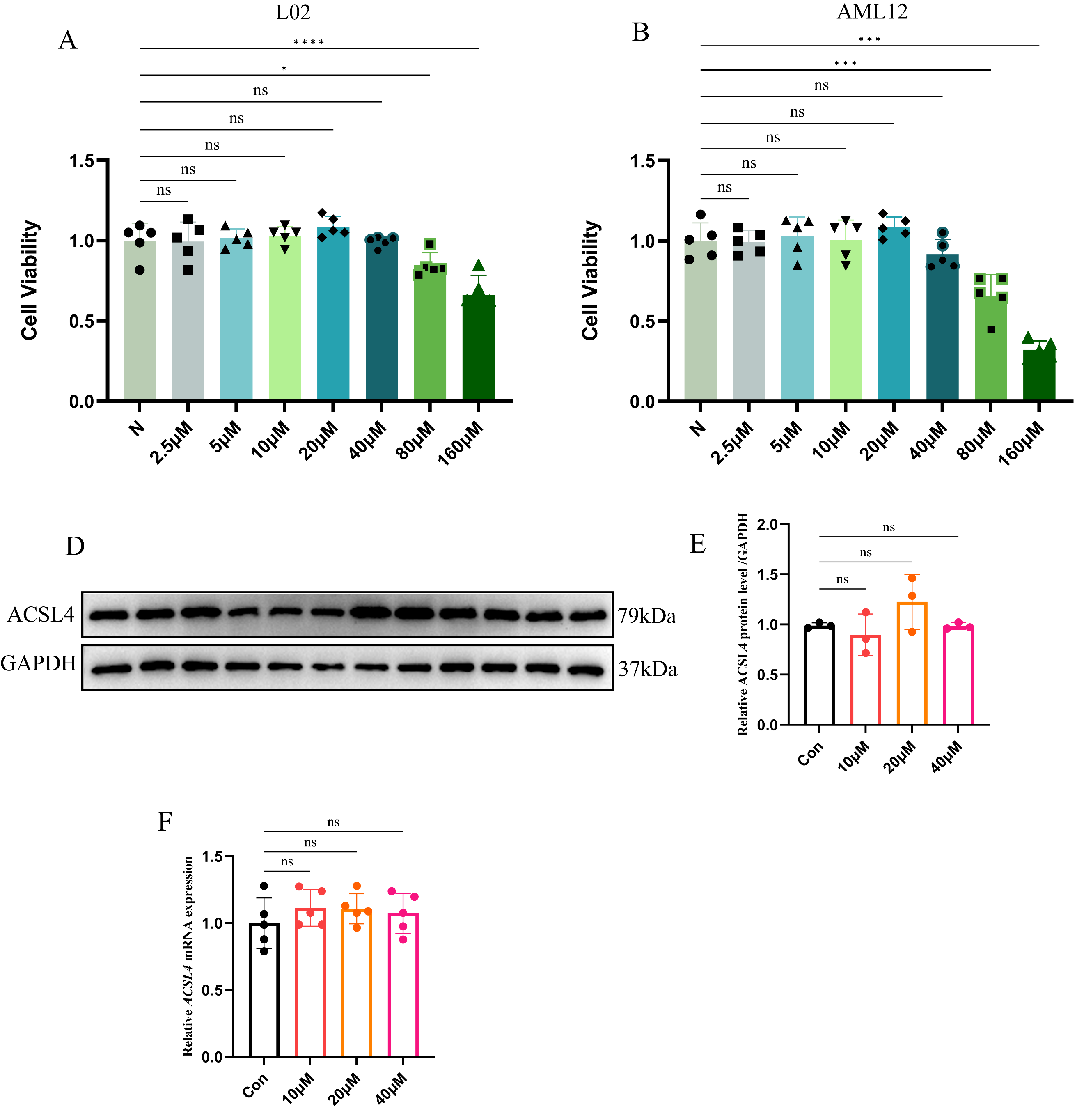
Figure S1**

**Effects of curcumin on hepatocyte toxicity and ACSL4 expression. (A)** Effects of different concentrations of curcumin on L02 viability of human hepatocytes. (B) Effect of curcumin on AML12 cell viability in mouse hepatocytes. (D-E) Immunoblot and semi-quantitative statistics of ACSL4 protein content in L02 cells under curcumin stimulation. (F) Effect of ACSL4 gene in L02 cells under curcumin stimulation. *P < 0.05, **P < 0.01, ***P < 0.001, ****P < 0.0001.
